# Inhibition of potato leafroll virus multiplication and systemic translocation by siRNA constructs against putative ATPase fold of movement protein

**DOI:** 10.1038/s41598-020-78791-4

**Published:** 2020-12-16

**Authors:** Priyanka Kumari, Jitesh Kumar, Ravi Ranjan Kumar, Mohammad Ansar, Kumari Rajani, Sunil Kumar, Tushar Ranjan

**Affiliations:** 1 Department of Molecular Biology and Genetic Engineering, Bihar Agricultural University, Sabour, Bhagalpur, 813 210 India; 2 Department of Plant Pathology, Bihar Agricultural University, Sabour, Bhagalpur, 813 210 India; 3 Department of Seed Science and Technology, Bihar Agricultural University, Sabour, Bhagalpur, 813 210 India; 4 Kusuma School of Biological Sciences, Indian Institute of Technology Delhi, Hauz Khas, New Delhi, 110016 India

**Keywords:** Plant biotechnology, Plant immunity, Plant molecular biology, Biotechnology

## Abstract

Viruses cause many severe plant diseases, resulting in immense losses of crop yield worldwide. Therefore, developing novel approaches to control plant viruses is crucial to meet the demands of a growing world population. Recently, RNA interference (RNAi) has been widely used to develop virus-resistant plants. Once genome replication and assembly of virion particles is completed inside the host plant, mature virions or sometimes naked viral genomes spread cell-to-cell through plasmodesmata by interacting with the virus-encoded movement protein (MP). We used the RNAi approach to suppress MP gene expression, which in turn prevented potato leafroll virus (PLRV) systemic infection in *Solanum tuberosum* cv. Khufri Ashoka. Potato plants agroinfiltrated with MP siRNA constructs exhibited no rolling symptoms upon PLRV infection, indicating that the silencing of MP gene expression is an efficient method for generating PLRV-resistant potato plants. Further, we identified novel ATPase motifs in MP that may be involved in DNA binding and translocation through plasmodesmata. We also showed that the ATPase activity of MP was stimulated in the presence of DNA/RNA. Overall, our findings provide a robust technology to generate PLRV-resistant potato plants, which can be extended to other species. Moreover, this approach also contributes to the study of genome translocation mechanisms of plant viruses.

## Introduction

Plant viruses cause serious damage to crops, and it is estimated that economic loss caused by viral pathogens ranks the second compared to that caused by other pathogens^1^. Potato leafroll virus (PLRV), a plant pathogen with a monopartite ssRNA genome, belongs to the family *Luteoviridae* and genus *Polerovirus*. It is found worldwide and is responsible for the loss of more than 20 million tonnes (up to 90%)^2^. These viruses infect potato plants most of the times in combination with other pathogens or sometimes individually. Since potato is vegetatively propagated, the chances of virus dissemination through progeny tubers are high. Tubers used for planting in the subsequent season can harbour latent viruses, which adversely affect plant growth and yield^3^.

Assembly of virion particles is a critical step during viral maturation process. In most plant viruses, assembly of virion particles occurs through the nucleation of capsid proteins (CPs) around nucleic acids^4^. The movement protein (MP) helps in either the passage of virion particles through plasmodesmata (PD) enlargement or direct transfer of genomic material in the form of nucleoprotein complex to the adjacent cells^5^. The mature plant virion particles spread to long distances by passing from one cell to another. They eventually infect a new host through vectors such as insects, nematodes, mites, and fungi. Targeting virus assembly or maturation process can be an effective strategy to develop PLRV resistance in plants. One of the most successful approaches to build plant resistance has been to introduce a part of the viral genomic sequence into the plant either to induce RNA silencing against viral RNA or to express intact or modified viral proteins or RNAs that disturb the viral infection cycle^6–8^. RNA interference (RNAi) technology has emerged as a potential tool to target virus assembly for developing resistant crops^6^. RNA silencing, a conserved regulatory mechanism of gene expression in eukaryotes, is triggered by dsRNA-provoking gene silencing through sequence-specific degradation of complementary mRNA transcripts (post-transcriptional gene silencing)^9^. Degradation of these target RNA occurs in a sequence-specific manner via formation of double-stranded RNA, which further processed into small interfering RNAs (siRNA) by the Dicer-Like (DCL) proteins and the RNA-Induced Silencing Complex (RISC)^10–12^.

Systemic infection of plant cells by viruses requires several crucial steps. After replication, plant virus particles spread to non-infected adjacent cells as virions or viral ribonucleoprotein (vRNP) complexes by cell-to-cell. The cell-to-cell movement of viruses through PD requires virus-encoded MPs^13^. MPs help in the enlargement of PD pore size and active transport of the viral nucleic acid into the adjacent cell, thereby allowing local spread of viruses in plants^14^. As MP plays a crucial role in PLRV transmission, we sought to analyse its amino acid sequence to identify conserved motifs and use them to develop a potential method of developing virus-resistant plants. First, we analysed the amino acid sequence of MP using bioinformatics tools. Our extensive bioinformatic analysis revealed four putative motifs, namely walker A, B, sensor, and arginine finger I and II, which possess ATP-binding and hydrolysis activities and are spread throughout the polypeptide chain of MP. We hypothesized that these motifs have a direct role in the active transportation of virions or nucleic acids through PD. To validate this hypothesis, we cloned, expressed, and purified MP in *Escherichia coli*. Further, we ascertained its function using nucleotide-stimulated ATP hydrolysis activity. Then, we conducted RNAi experiments against MP gene to check its effects on PLRV infection. As MP homologues are present in most plant viruses, their inhibition via RNAi can be a potential method for generating virus-resistant plants.

## Materials and methods

### Sequence analysis, motif identification, and structural analysis

The sequences of MP from different strains of PLRV were retrieved from the NCBI database (http://www.ncbi.nlm.nih.gov/). Multiple sequence alignments were generated using ClustalW^15^ and were manually corrected for domain superimpositions. Atomic structure of MP was predicted using Iterative Threading ASSEmbly Refinement (I-TASSER), a tool which builds atomic structures on the basis of iterative template fragment assembly simulations and multiple-threading alignment using Locally Installed Meta-Threading Server (LOMETS). This prediction returns structures with good C-scores in the range of − 4.28 to − 2.17^16^.

### Cloning, expression, and purification of PLRV_MP (ORF4)

Total RNA was extracted from infected potato leaves, and cDNA of PLRV was prepared using reverse transcriptase PCR. This cDNA was used as template for the PCR amplification of MP (FP: 5′-CCGG*AAGCTT*ATGTCAATGGTGGTGTACAACAACC-3′; RP: 5′-GCGC*CTCGAG*TCATCCGCGCTTGATAAGTTTTGG-3′). The amplified DNA fragments were concentrated by ammonium acetate precipitation, digested with appropriate restriction enzymes (HindIII and XhoI), and purified by agarose gel electrophoresis and gel extraction using manufacturer’s protocols. The digested fragment was ligated to the pET28b plasmid, which was linearized with the same restriction enzymes. The insertion of the MP gene in the plasmid leads to the fusion of 6× histidine tag at the N-terminus of the gene. The ligated DNA was transformed into *E. coli* DH5α competent cells, and plasmid DNA was prepared from successful recombinants using the alkaline lysis method. The accuracy of the cloned DNA inserts was further confirmed by sequencing. Finally, the MP recombinant plasmids were transformed into *E. coli* BL21 (RIPL) cells, and MP expression was induced with 0.5 mM IPTG at 30 °C for 3 h. For the purification of the recombinant protein, the induced cells were harvested by centrifugation at 5800×*g* for 15 min at 4 °C and were lysed using a sonicator at 40% amplitude (2 s on/1 s off for 20 min). The cell lysate was centrifuged at 17,000×*g* for 45 min at 4 °C, and the supernatant and pellet were separated. The supernatant containing the protein of interest was loaded onto pre-equilibrated Ni–NTA beads (ThermoFisher Scientific) and the His-tagged protein was eluted using 500 mM imidazole. Elution fractions containing the protein of interest were identified and confirmed by SDS-PAGE analysis.

### Malachite green-based ATPase assay

Colorimetric detection of inorganic phosphate (Pi) was performed using a malachite green-based method^17^. The malachite green reagent (0.14% malachite green, 7.5% ammonium molybdate, 11% Tween-20 in 5 N H_2_SO_4_) was used to detect free Pi. In this method, Pi reacts with molybdate and forms a green-coloured complex, which can be quantified at 630 nm. The purified MP at two different concentrations of 2 and 10 µM was mixed with 2 mM ATP in a 20 µL buffer containing 20 mM Tris–HCl (pH 8.0) and 2 mM MgCl_2_. The reaction mixture was incubated at 37 °C for 30 min. The reaction was stopped by adding 50 mM EDTA. Two hundred microliter of the malachite green reagent was added to the reaction mixture, and Pi generated through ATPase activity was quantified at 630 nm. Similar experiment was also performed in the presence of dsDNA (471 bp; 40 ng) and RNA as well (40 ng). A standard curve was generated using 0–20 μΜ KH_2_PO_4_ as a source of phosphate.

### Gel-shift assay

Binding reactions (20 µL) containing DNA substrate (40 ng) and increasing amounts of protein (0.5–10 µM) were incubated along with 2 mM ATP for 30 min at 37 °C in a buffer containing 20 mM Tris–HCl (pH 8.0), 5 mM MgCl_2_. The MP-DNA mixture were loaded onto a 0.8% agarose gel and were separated by electrophoresis in 0.5× TAE buffer at 90 V for 2 h at room temperature. The complexes were visualized in UV light.

### Preparation of siRNA constructs

To generate target-specific siRNAs against MP gene, MP encoding gene fragments amplified from the cDNA of PLRV as template and digested with XhoI and KpnI were cloned into the pHANNIBAL vector at XhoI–KpnI sites in sense orientation (pHANNIBAL-sense MP). The primer sequence for cloning of MP encoding gene in sense direction were designed manually and synthesized from IDT, USA. The details of which are as follows: FP: 5′-CCGG*CTCGAG*ATGTCAATGGTGGTGTACAACAACC-3′; RP:5′-GCGC*GGTACC*TCATCCGCGCTTGATAAGTTTTGG-3′. The pHANNIBAL vector contains the CaMV 35S promoter and the NOS terminator in sense orientation. Subsequently, the amplified and digested anti-sense MP gene fragments were cloned into the same vector at HindIII–BamHI sites to generate antisense construct (pHANNIBAL-antisense MP). The details of primer sequences are as follows: FP: 5′-GGGT*AAGCTT*TCATCCGCGCTTGATAAGTTTTGGC-3′; RP: 5′-GCGC*GGATCC*ATGTCAATGGTGGTGTACAACAAC-3′. The resulting siRNA construct containing the sense and anti-sense fragments of the MP cDNA sequence was named as pHANNIBAL-MP. The siRNA cassettes were released from pHANNIBAL-MP by NotI digestion and introduced into the binary vector pART27 to form the siRNA construct pART27-MP for plant transformation.

### Transient expression assay

The binary plasmids pART27-MP and the empty vector pART27 were extracted and purified from *E. coli* cultures and were transformed into the *Agrobacterium tumefaciens* EHA101 using the freeze–thaw transformation method^18^. The transformed cells of *A. tumefaciens* were plated on Luria–Bertani (LB) agar plates containing 50 μg/mL kanamycin, 100 μg/mL spectinomycin, and 100 μg/mL chloramphenicol for plasmid selection. Transformation of the binary plasmids was further confirmed using colony PCR for the MP gene. For agroinfiltration, *A. tumefaciens* cells harbouring the pART27-MP siRNA constructs were grown overnight at 28 °C in the LB medium supplemented with appropriate antibiotics. The overnight cultures were diluted 1:10 in in fresh media containing the above-mentioned antibiotics, 10 mM 2-(*N*-morpholino) ethanesulfonic acid (MES), and 200 μM acetosyringone to an OD_600_ of 0.3. The cells were collected by centrifugation at 5000×*g* for 5 min and were resuspended in the infiltration medium containing 10 mM 2-(*N*-morpholino) ethanesulfonic acid (MES), 10 mM MgCl_2_, and 200 μM acetosyringone. The cells were then incubated at room temperature for 2–3 h before agroinfiltration. *S. tuberosum* cv. Khufri Ashoka plants were agroinfiltrated with 2 mL of syringe directly into the phloem and leaf as well. Whole plants were covered with a transparent plastic bag for 3–4 days. All experiments were repeated thrice with five plants in each experiment for each siRNA constructs and control.

### Viral infectivity assay

Newly emerged symptomatic potato plants from PLRV infected tubers were used for agroinfiltration of pART27-MP siRNA constructs. PLRV infected tubers were grown in a controlled environment. The presence of PLRV titre in tubers and newly emerged plants were confirmed through ELISA and CP specific primers (FP: 5′ATGAGTACGGTCGTGGTTAGA-3′; RP: 5′ CTATCTGGGGTTCTGCAAAGCC-3′). The plants were grown in a transgenic glass house at 25 °C with 16 h of light and 8 h of darkness after agroinfiltration. Ten days post agroinfiltration, newly tertiary leaves were harvested for RNAi analysis. The RNAi constructs were analysed using Northern blotting, RT-PCR and enzyme-linked immunosorbent assay (ELISA).

### Northern blotting analysis of siRNA

Agroinfiltrated potato plant and control healthy plant leaves were used to isolate small RNAs from 5 and 10 dpi plants using PAX gene Tissue RNA/miRNA Kit (Qiagen,Germany) following the manufacturer’s protocol. The denatured polyacrylamide gels (15%) containing 7 M urea were run at 40 mA (600 V) for approximately 2 h to resolve the total RNA containing the siRNA pool. The gels were stained for 10 min with 0.5 μg/ml EtBr in DEPC-treated TBE buffer. A Bio-Rad transblot apparatus (Bio-Rad, USA) was used to transfer the RNA onto Hybond-N + positively charged nylon membranes (GE healthcare, USA) at 200 mA (9–10 V) for 3 h followed by crosslinking for 20 min at 1200 μjoules and drying for 30 min at 50 °C to improve sensitivity. Further, membrane was pre-hybridized in pre-hybridization buffer (7% SDS, 200 mM Na_2_HPO_4_ (pH 7.0), 5 μg/ml salmon sperm DNA (SSDNA)) at 40 °C for 30 min, which was then replaced with hybridization buffer containing biotin-labeled probes at 50 pmol/ml concentration, the details of which are provided in supplementary file (Table S1). The membranes were subsequently hybridized at 40 °C for 12 to 16 h with continuous gentle shaking followed by rinsing with washing buffer (1× SSC, 0.1% SDS) thrice for a total of 15 min at room temperature. The membrane was then blocked for 15 min with blocking buffer using gentle shaking at room temperature followed by incubation with hybridization buffer containing stabilized streptavidin-HRP conjugate for an additional 15 min. The blot was visualized using Amersham typhoon blot imaging systems (GE, Healthcare) after developing by ECL (GE Healthcare).

### RT-PCR analysis of viral RNA in potato plants

Total RNA was extracted from the newly tertiary leaves (100 mg) of agroinfiltrated potato plants and control healthy plants using RNeasy Plant Mini kit (Thermo Scientific, USA) following manufacturer’s instructions. RT-PCR was conducted for MP-infiltrated plants individually using the PLRV-specific primers. cDNA was synthesized using RT-PCR kit (Thermo Scientific, USA). Total reaction volume of 20 μL contained the RNA template, sterile water, 10 mM dNTPs, and 50 μM random nanomers. Initially, the reagents were incubated at 70 °C for 10 min; subsequently, sterile water, 10× RT buffer, 20 U/μL RNase inhibitor and 20 U/μL enhanced AMV-RT were added, and the mixture was incubated further at 25 °C for 15 min and at 45 °C for 50 min. The total reaction mixture volume for PCR was 50 μL.

### ELISA

Indirect ELISA was performed for the detection of PLRV infection. Tertiary leaves of agroinfiltrated plants and control leaves (positive and negative) of potato (0.5 g) were macerated in 5 mL extraction buffer containing 0.05 M phosphate-buffered saline and 0.01 M sodium diethylene carbamide (pH 7.4). As positive and negative controls, PLRV infected and uninfected/healthy potato leaves were considered, respectively. The extracts were centrifuged at 10,000×*g* for 5 min. One hundred microliter of the supernatant was added to the ELISA plate, which was incubated overnight at 4 °C. Further, 200 μL of rabbit polyclonal antibody against PLRV-CP (1:5000; Promega) was added to each well and incubated for 3 h at 37 °C. After washing, goat anti-rabbit immunoglobulin G conjugated with alkaline phosphatase (Promega, USA) was added to the wells and incubated for 3 h at 37 °C. The substrate p-nitrophenyl phosphate was added to the plate for developing and absorbance at 405 nm was measured. The absorbances of samples and controls were normalized to that of the positive control. The data were statistically analysed and the significant differences between means were calculated by one way and two way ANOVA (p < 0.001).

## Results

### The putative ATPase motifs

**Figure 1:**
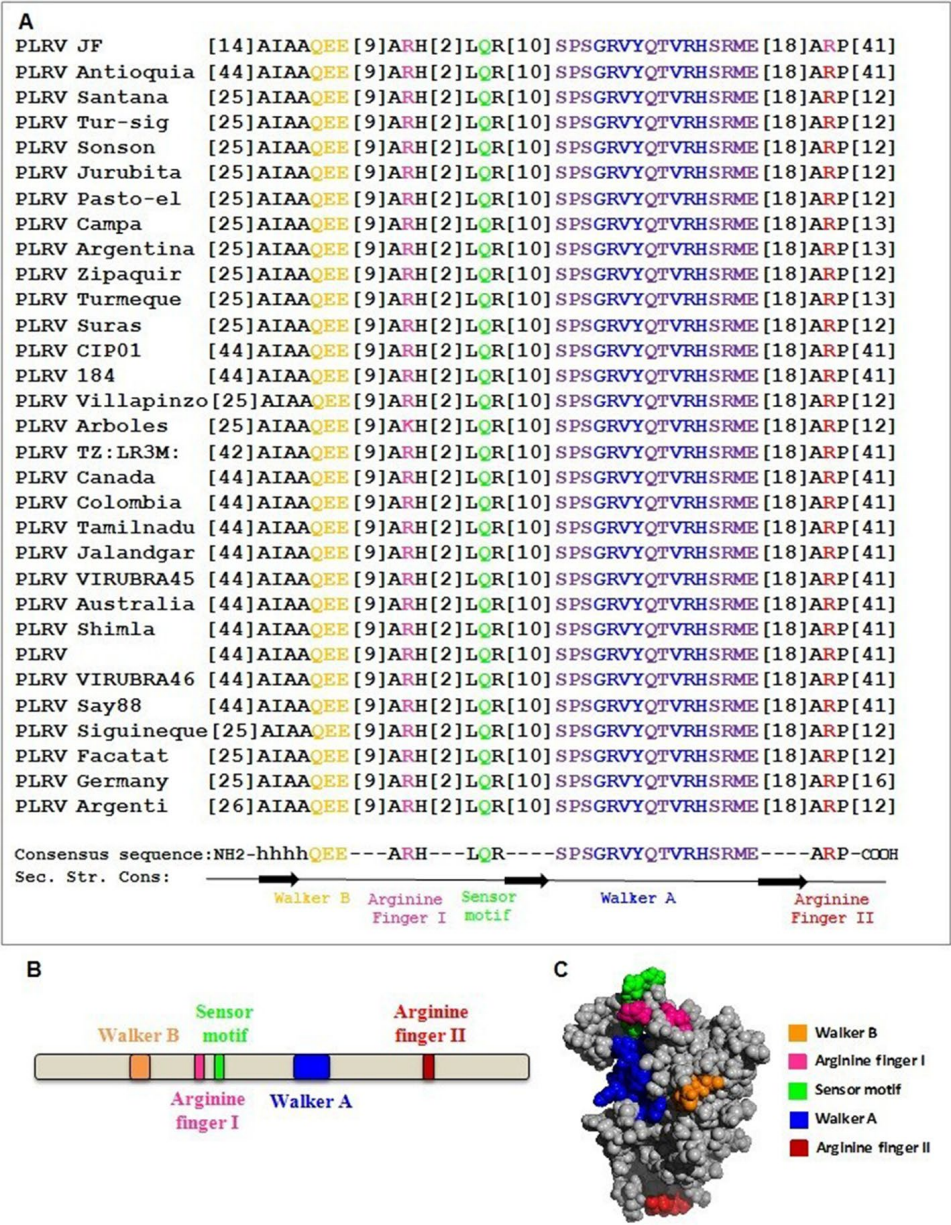
(**A**) Sequence alignments of the ATPase domain of PLRV_MP. Multiple sequence alignments, generated using ClustalW, were manually corrected for domain superimpositions. The number(s) in brackets represent the number of amino acids. (**B**) Organization of functional motifs on the polypeptide chain of MP of PLRV. Schematics are drawn approximately to the scale and represent the approximate consensus of representative homologs. (**C**) I-TASSER predicted atomic model of PLRV_MP with all the putative ATP catalysis motifs. [Accession Numbers: Potato leafroll virus isolate PLRV-JF (PLRV JF): ADE93562.1, Potato leafroll virus isolate Anoquia (PLRV Antioquia): QES86451.1, Potato leafroll virus isolate Sta Rosa de Osos-santana (PLRV Santana): AEB78381.1, Potato leafroll virus isolate Turmeque-siguineque (PLRV Tur-sig): AEB78353.1, Potato leafroll virus isolate Sonson-tasajo (PLRV Sonson): AEB78367.1, Potato leafroll virus isolate Siachoque-jurubita (PLRV Jurubita): AEB78379.1, Potato leafroll virus isolate Pasto-el campanero2 (PLRV Pasto-el): AEB78377.1, Potato leafroll virus isolate Pasto-el campanero (PLRV Campa): AEB78375.1, Argentinian Potato leafroll virus isolate PLRV-5 (PLRV Argentina): ADE93558.1, Potato leafroll virus isolate Zipaquira-san Jorge (PLRV Zipaquir): AEB78361.1, Potato leafroll virus isolate Turmeque-siguineque2 (PLRV Turmeque): AEB78373.1, Potato leafroll virus isolate Ipiales-suras (PLRV Suras): AEB78385.1, France Potato leafroll virus strain CIP01 (PLRV CIP01): AAL77949.1, Potato leafroll virus isolate PLRV184 (PLRV 184): AYA73306.1, Potato leafroll virus isolate Villapinzon-bosabita (PLRV Villapinzo): AEB78359.1, Potato leafroll virus isolate Madrid-los arboles2 (PLRV Arboles): AEB78369.1, Potato leafroll virus isolate TZ:LR3M:11 (PLRV TZ:LR3M): AGN48055.1, Potato leafroll virus isolate PLRV171 (PLRV Canada): AYA73300.1, Colombia Potato leafroll virus isolate PLRV165 (PLRV Colombia): AYA73294.1, Tamil nadu Potato leafroll virus isolate OTNI-2 (PLRV Tamilnadu): AFJ11889.1, Jalandgar Potato leafroll virus isolate (PLRV Jalandgar): AFJ11863.1, Karlovce Potato leafroll virus isolate VIRUBRA 1/045 (PLRV VIRUBRA45): ACD93696.1, Australia Potato leafroll virus (PLRV Australia): QBO24572.1, Shimla Potato leafroll virus isolate PBI-6 (PLRV Shimla): AFJ11881.1, Potato leafroll virus (PLRV): QBO24572.1, Prague Potato leafroll virus isolate VIRUBRA 1/046 (PLRV VIRUBRA46): ACD93705.1, Potato leafroll virus isolate Say88 (PLRV Say88): QBO24572.1, Potato leafroll virus isolate Turmeque-siguineque3 (PLRV Siguineque3): AEB78383.1, Potato leafroll virus isolate Facatativa (PLRV Facatat): AEB78357.1, Germany Potato leafroll virus isolate PLRV-DSMZ (PLRV Germany): ADE93559.1, Argentinian Potato leafroll virus isolate PLRV-5 (PLRV Argentinian): ADE93558.1].

MPs are known as plant virus-encoded factors that interact with PD to mediate the intercellular spread of viruses in planta. All the putative motifs necessary for ATP binding and hydrolysis are present in the middle domain of the viral MP, except Walker B (orange) and arginine finger II (red) which are situated at N and C-terminus respectively (Fig. 1A,B). The Walker A motif (blue), with the consensus sequence GRVYQTVRH, interacts with the β and γ-phosphates of the bound nucleotide, whereas the conserved Glu of Walker B (consensus sequence hhhhQEE), located next to a β-strand, binds to a metal ion and helps in ATP hydrolysis. The Walker-B Glu coordinates the Mg^2+^ cation, while another conserved catalytic Glu residue primes a water molecule for the nucleophilic attack on the γ-phosphate of the bound ATP. During our multiple sequence alignment analysis, we noted that the MP sequences from all PLRV strains showed the presence of a highly conserved hydrophobic residue before the catalytic Glu and the overall Walker B motif (orange), indicating that MPs related to ASCE P-loop ATPases (Fig. 1A). Further, we observed that the Arginine finger I (pink) and II motifs (red), which are located 9–10 residues downstream of Walker B motif and 18 residues downstream of Walker A, and the sensor motif (green) present about two residues further downstream of the Arginine finger I motif are strictly conserved across the strains of PLRV analysed in this study (Fig. 1A). Our structure prediction using I-TASSER revealed that the active site of PLRV MP comprises all the motifs necessary for ATP interaction except Arginine finger II, which is situated far from the rest of the motifs in their folded structure (highlighted in red; Fig. 1C). Arginine finger is a classical hallmark of ATPases and is conserved across many ATPases. It completes the active site from a distinct location, forming contacts with the γ-phosphate of the nucleotide^19^.

### Expression and purification of PLRV_MP

**Figure 2:**
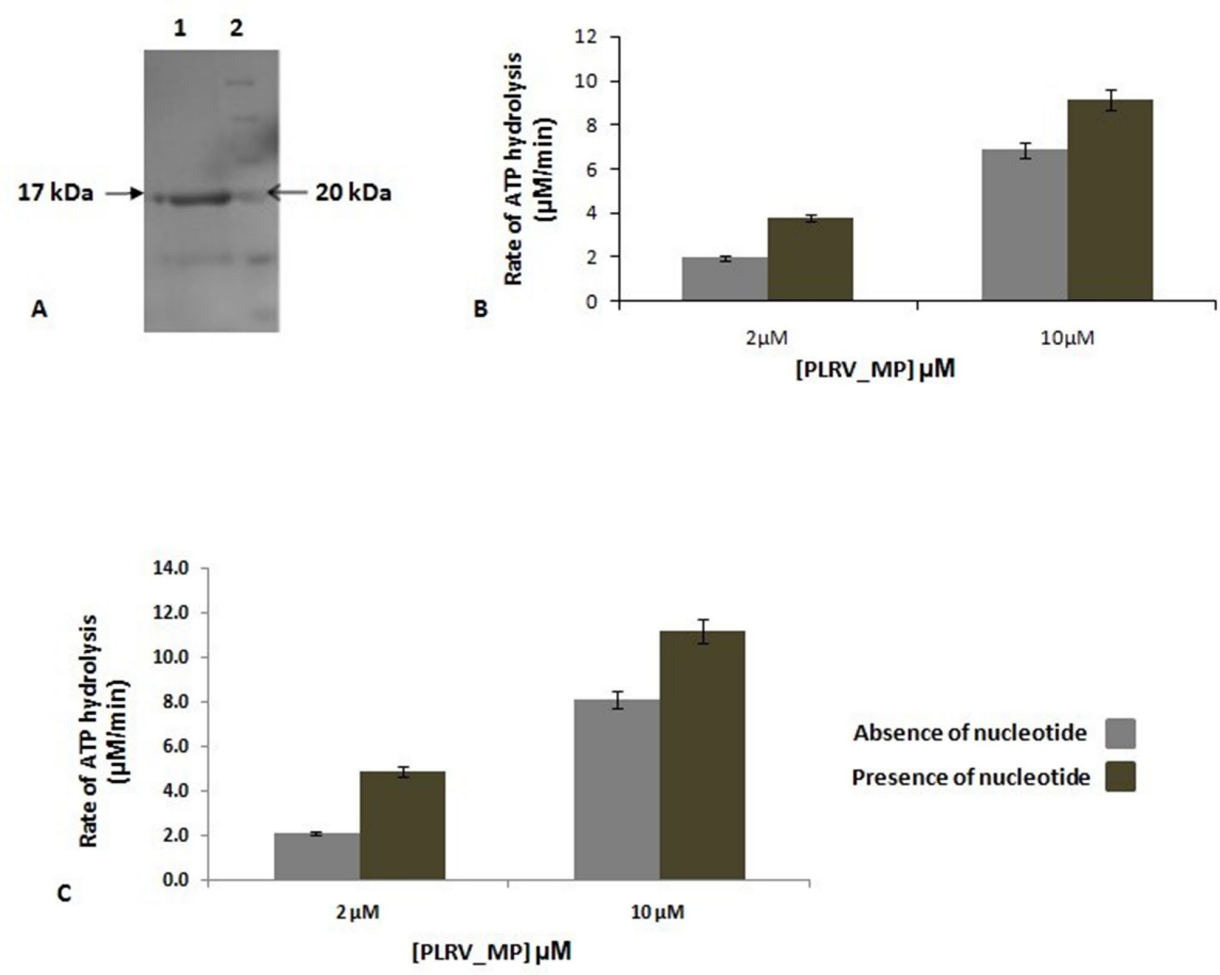
(**A**) SDS-PAGE analysis of affinity purified his-tagged PLRV_MP expressed in *E. coli*. Lane 1: Purified protein and lane 2: Marker. (**B**) Purified MP exhibits basal and DNA-stimulated ATPase activity. (**C**) Apart from this, MP also exhibits RNA stimulated ATPase activity. Protein concentrations were 2 μM and 10 μM. Only protein is indicated with light grey and protein + dsDNA is indicated with dark grey. The vertical bars represent the mean values of three replicates (n = 3) and the values are highly significant exhibited at 0.001 level. DNA/RNA concentration was maintained constant at 40 ng for every independent reaction. Negative controls with only buffer were also taken into account while performing the reactions.

The PLRV MP was cloned in pET28b, expressed in *E. coli*, and purified using affinity chromatography. The purified protein was analysed using SDS-PAGE; the single prominent band at ~ 20 kDa indicates successful purification of the protein (Fig. 2A).

### DNA/RNA stimulates the ATPase activity of purified MP by twofold

As the ATPase domain of MP is believed to be the driving force for DNA transportation across PD, the ability of the purified protein to hydrolyse ATP was analysed (Fig. 2A,B). Malachite green-based ATPase assay was performed to test the function of the purified MP in the presence of dsDNA and ssRNA. The ATPase activity was assayed by maintaining the amount of ATP at 2 mM and having two different concentrations of MP: 2 and 10 µM. With increasing protein concentrations, the rate of ATP hydrolysis increased (Fig. 2B; light grey). In addition, the ATPase activity of the purified MP was further enhanced in the presence of dsDNA (450 bp; 40 ng) (Fig. 2B; dark grey) and ssRNA (40 ng) (Fig. 2C; dark grey). The nucleotide binding evidence of recombinant MP was further confirmed by gel shift assay (Fig. S1).

### Construction of the pART27 binary vector

**Figure 3:**
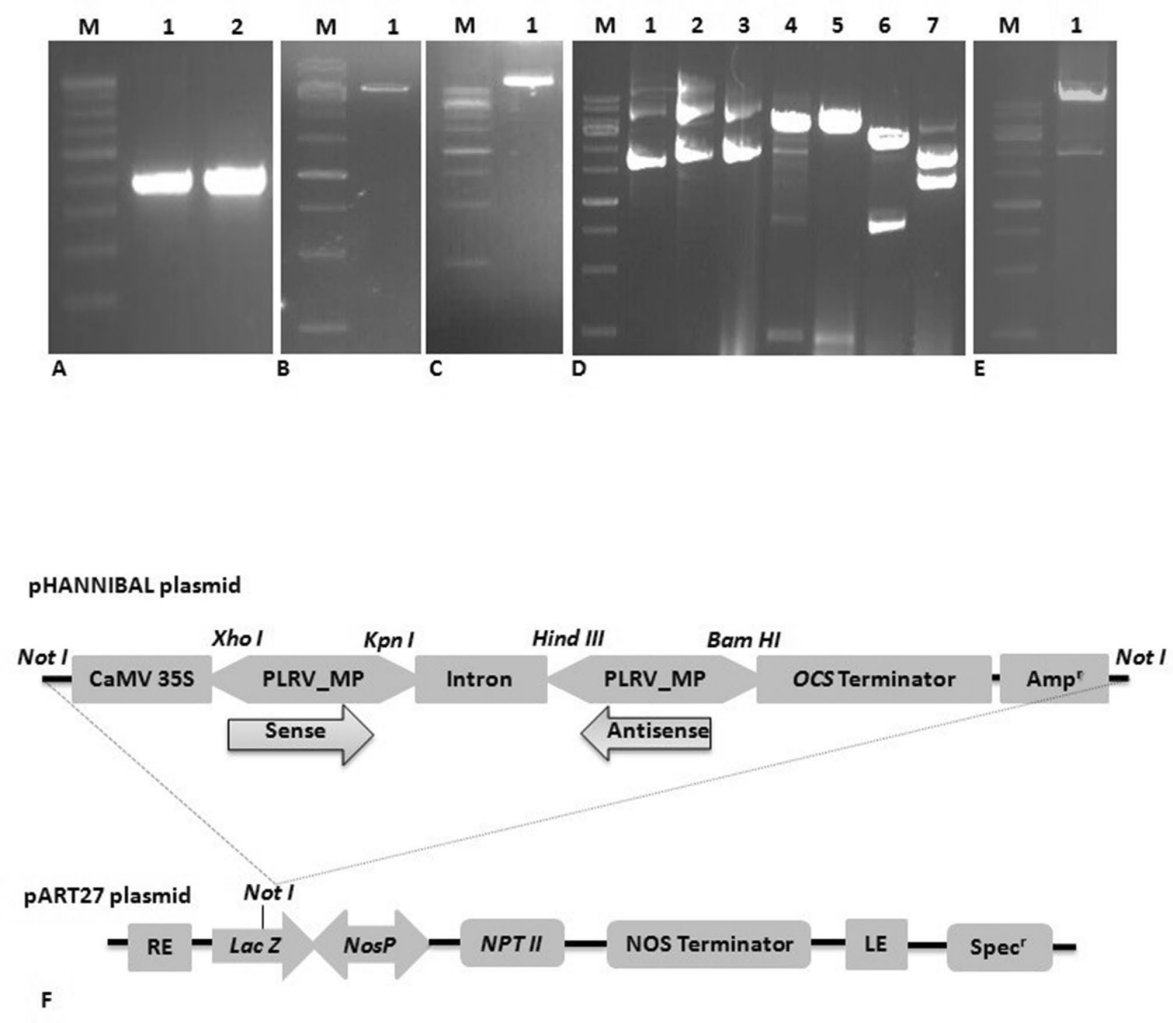
Reverse-transcription polymerase chain reaction (RT-PCR) was performed to amplify sense and antisense PLRV_MP sequences from the leaves of infected potato plants. (**A**) Amplified cDNA fragments were analyzed by electrophoresis on 0.8% agarose gel. M: 100 bp DNA ladder; lanes 1 and 2: PCR products of sense and antisense MP (471 bp). (**B**) The pHANNIBAL plasmids were purified from *E. coli* DH5a cultures and analyzed by electrophoresis on 0.8% agarose gel. M: 1 kb DNA ladder; lanes 1: pHANNIBAL plasmid (5.83 kb). (**C**) The pART27 binary plasmid was purified from E. coli DH5a culture and analyzed by electrophoresis on 0.8% agarose gel. M: 1 kb DNA ladder; lane 1: pART27 plasmid (11.6 kb). (**D**) Confirmation of siRNA constructs in pHANNIBAL and pART27 binary vector through restriction analysis. M: 1 kb DNA ladder; lane 1: undigested pHANNIBAL; lane 2: antisense MP ligated with pHANNIBAL (pHANNIBAL-antisense MP construct); lane 3: both antisense and sense MP ligated with pHANNIBAL (pHANNIBAL-MP-siRNA construct); lane 4: HindIII and BamHI digestion of pHANNIBAL- antisense MP construct shows the release of a 471 bp fragment; lane 5: XhoI and KpnI digestion of pHANNIBAL-MP siRNA construct also shows the release of 471 bp fragments; lane 6: XhoI and BamHI digestion of pHANNIBAL-MP siRNA construct shows the release of a ~ 1500 bp fragment; and lane 7: NotI digestion of pHANNIBAL-MP siRNA construct shows the release of two fragments of ~ 4 kb (including sense MP, intron, and antisense MP sequence) and 3.5 kb. (**E**) The whole siRNA cassette (~ 4 kb) was transferred from pHANNIBAL to pART27, and this was further confirmed by NotI restriction analysis. (**F**) Schematic representation of pART27-MP siRNA constructs used for transient expression by agroinfiltration.

Specific primers were designed for the amplification of the gene sequence of MP in sense and antisense orientation. The total RNA extracted from infected potato leaves was subjected to RT-PCR using these primers and the desired sequences were amplified. PCR amplification of an oligo dT-primed first-strand cDNA template prepared from the mRNA extracted from infected leaves yielded 471 bp bands of both sense and antisense orientation (Fig. 3A). These fragments were cloned into the pHANNIBAL vector and digested with the appropriate set of enzymes (Fig. 3B). The cloning of the MP gene in pHANNIBAL in sense orientation (pHANNIBAL-sense MP) and antisense orientation (pHANNIBAL-antisense MP) was carefully analysed with different sets of restriction enzymes (Fig. 3D; lanes 1–7). Further, the orientation of genes was confirmed through sequencing (IDT). Another round of sequencing was also performed in cloning vector carrying both sense and antisense MP (pHANNIBAL-MP). The resulting siRNA constructs were further digested by NotI and introduced into the digested linear binary vector pART27 to generate the pART27-MP-siRNA construct (Fig. 3C–F). The accuracy of siRNA constructs in pHANNIBAL and pART27 binary vector was further confirmed through restriction digestion analysis (Fig. 3D).

### Transient expression of MP siRNA

**Figure 4:**
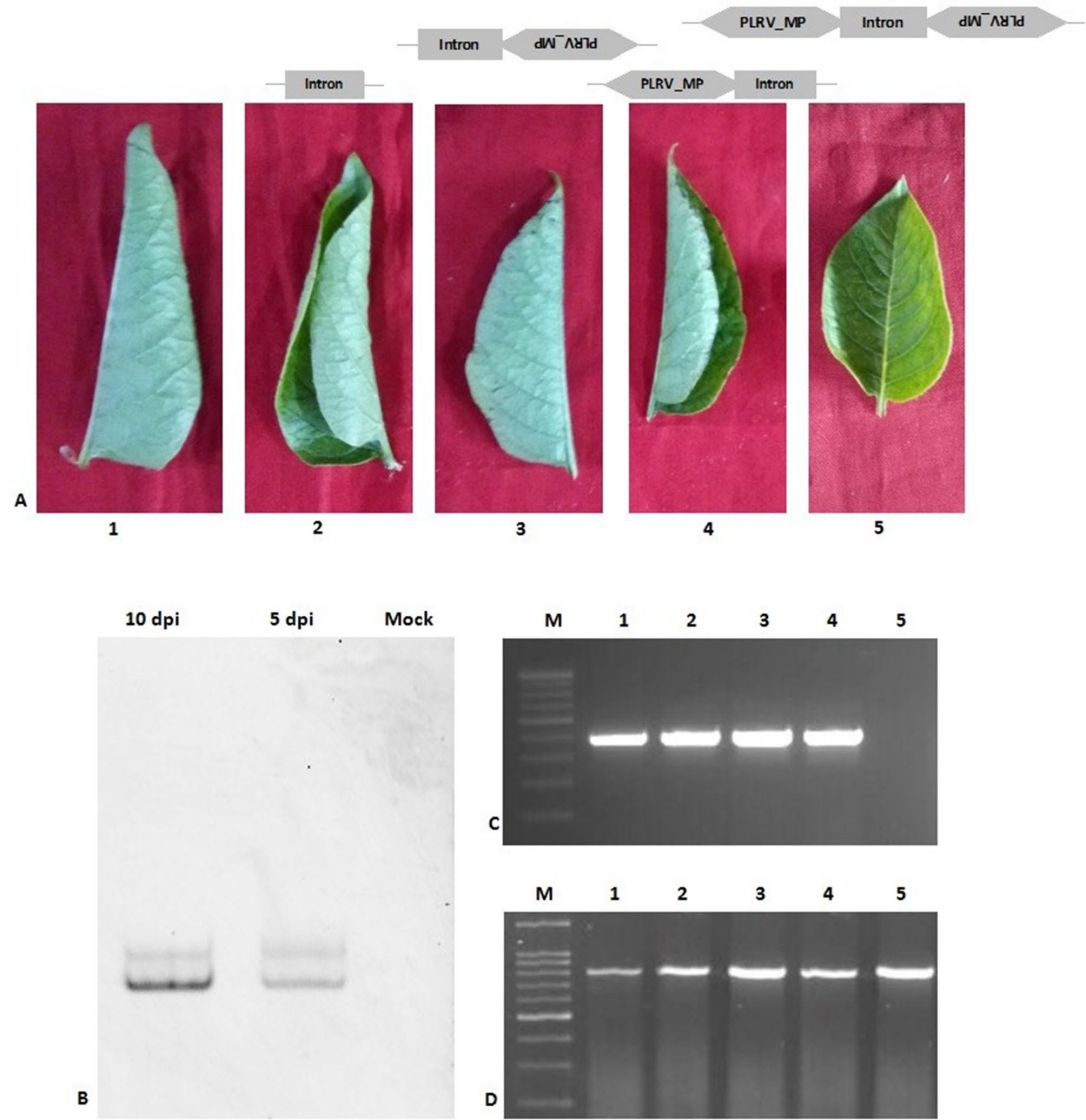
(**A**) Symptoms observed in the tertiary leaves of the PLRV infected potato plants. (1) PLRV infected control without agroinfiltration; (2) agroinfiltrated with the empty vector pART27; (3) agroinfiltrated with the plasmid containing only antisense sequence (pART27-antisense MP); (4) agroinfiltrated with the plasmid containing only sense sequence (pART27-sense MP); (5) agroinfiltrated with the pART27-MP siRNA construct; (**B**) Confirmation of expression of siRNA using Northern blotting analysis. Higher expression of siRNA in the leaves at 10 dpi (lane 1) was observed as compared to 5 dpi (lane 2), whereas empty vector (mock) agro-infected leaves did not show any siRNA (lane 3). (**C**) Detection of PLRV RNA by RT-PCR. Amplified cDNA fragments (627 bp) were analyzed by electrophoresis on 0.8% agarose gel. M: 100 bp marker; lane 1: PLRV was observed in control plant; lane 2: PLRV from tertiary leaves of the plant containing the empty vector pART26; lane 3: PLRV from tertiary leaves of the plant containing the antisense construct (pART27-antisense MP); lane 4: PLRV from tertiary leaves of the plant containing the sense construct (pART27-sense MP); lane 5: no PLRV in tertiary leaves of the plant containing MP siRNA (pART27-MP). (**D**) Actin PCR was performed as an internal control.

To determine whether the knockdown of MP could suppress the systemic translocation of PLRV in potato, the pART27-MP-siRNA constructs were agroinfiltrated into *S. tuberosum* cv. Khufri Ashoka. Control plants (PLRV infected) (Fig. 4A; lane 1) and those agroinfiltrated with the empty vector (Fig. 4A; lane 2) showed rolling symptoms of PLRV infection in the upper leaves, whereas plants that were agroinfiltrated with the MP siRNA constructs (pART27-MP) did not show any symptoms of viral infection (Fig. 4A; lane 5). Rolling symptoms were also observed in the upper leaves of plants agroinfiltrated with pART27-sense MP and pART27-antisense MP construct (Fig. 4A; lanes 3 and 4 respectively). These findings indicated that siRNA constructs hampered or suppressed viral translocation and multiplication in the plants.

### Analysis of agroinfiltrated potato plants harbouring siRNA constructs

The expression of siRNA was analysed by Northern blotting using an equal amount of total RNA from the leaf samples of pART27-MP construct and empty vector harbouring plants respectively, to ascertain whether RNA silencing can be initiated in agro-infected potato leaves by pART27-MP construct. The agro-infected regions of the leaf were used to isolate total RNA which was analysed for the accumulation of siRNAs 5 and 10 days after inoculation. The siRNAs were detected in the agro-infected region from day 5, 10 (Fig. 4B). The RNA band intensity suggested higher expression of siRNA in the leaves at 10 dpi (Fig. 4B; lane 1) as compared to the ones at 5 dpi (Fig. 4B; lane 2), whereas empty vector (mock) agro-infected leaves did not show any presence of siRNA (Fig. 4B; lane 3).

### RT-PCR analysis of CP gene

To detect the presence of the virus in the host tissue, total RNA isolated from the upper tertiary leaves of agroinfiltrated plants and control plants was subjected to RT-PCR using PLRV-specific primers. The 627 bp amplified DNA fragment corresponding to the CP gene of PLRV was detected in the tertiary leaves of plants agroinfiltrated with the empty vector and control leaves; however, it was not detected in the tertiary leaves of plants agroinfiltrated with the MP siRNA constructs. Thus, RT-PCR confirmed that PLRV infection was suppressed by these siRNA constructs (Fig. 4C). RT-PCR for the actin gene was also performed simultaneously as an internal control (Fig. 4D).

### ELISA to detect PLRV in host plants

**Figure 5:**
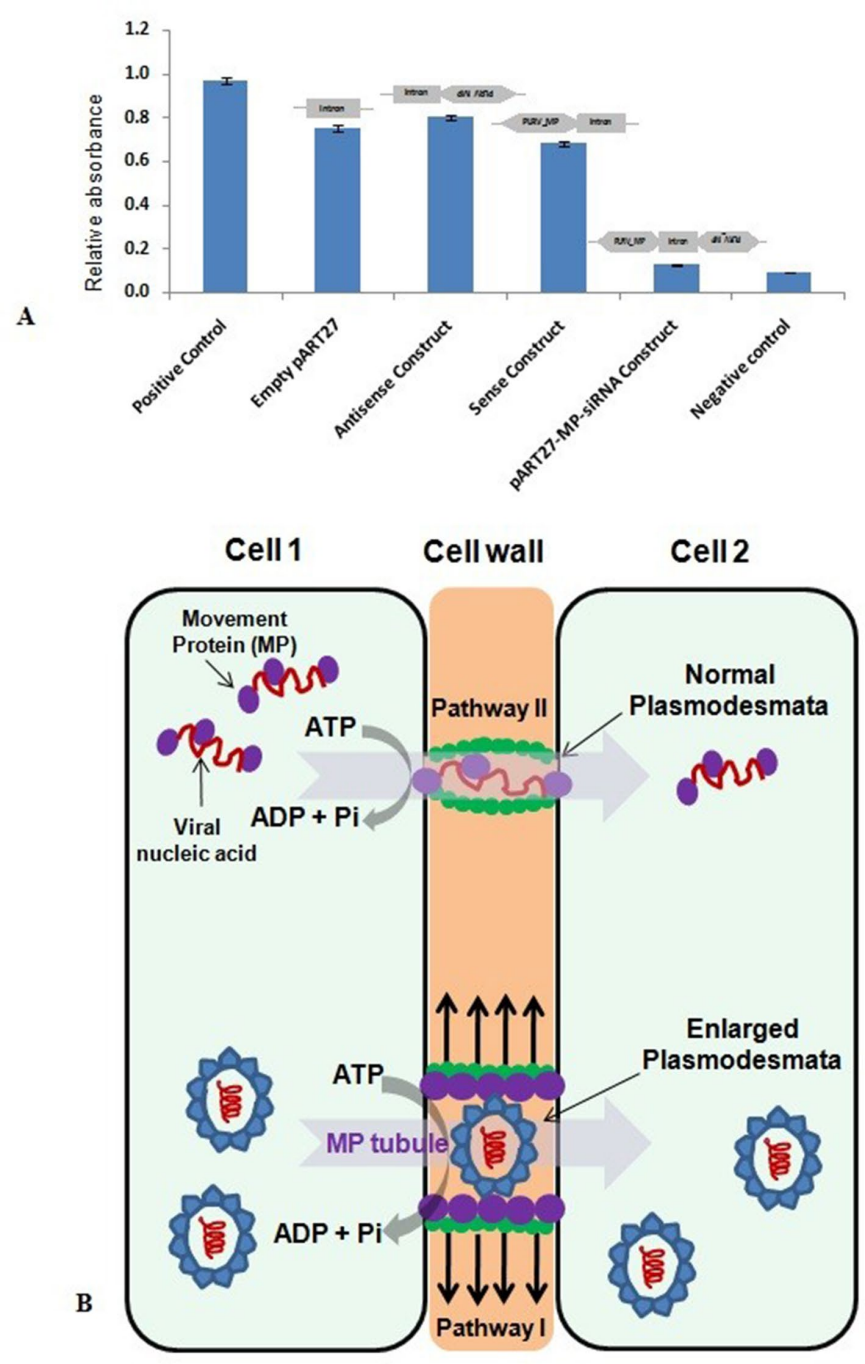
(**A**) Absorbance values of all the samples were normalized to that of the positive control. The vertical bars represent the mean ± S.E. of three replicates (n = 3) and the values are highly significant exhibited at 0.001 level. (**B**) Hypothetical model for the transportation of mature virion particles (pathway I; lower panel) and their genomes through plasmodesmata with the assistance of ATP hydrolysis by MP (pathway II; upper panel).

The presence of PLRV in host plants was also detected by DAS-ELISA using anti-PLRV CP antibody. The absorbance values of all the samples were normalized to that of the positive control. The mean absorbance values for all the samples are presented in Fig. 5A. We noted that the relative absorbance values of the samples from plants containing the siRNA constructs were two times that of the negative controls. Overall, PLRV CP was undetectable in plants agroinfiltrated with siRNA, whereas PLRV was present in high concentration in the plants agroinfiltrated with the empty vector.

## Discussion

Plant viruses cannot exploit membrane fusion-based routes of entry as described for animal viruses. Once plant viruses enter the cell by means of a vector, they need to enhance the restricted pore size of PD (connecting channels between plant cells). Thus, the transportation of virion particles through PD from one cell to another cell is a critical step during infection of plant viruses (Fig. 5A, pathway I; lower panel). Previous studies have revealed that PD allow the cell-to-cell trafficking of plant viruses with the help of viral encoded MPs^20–22^. In addition, MPs are also involved in PD gating that allows the direct intercellular movement of viral genomes (Fig. 5B, pathway II; upper panel). Various host factors such as cytoskeleton, endoplasmic reticulum, and other endomembrane interact with MPs to regulate complex mechanisms of PD gating^20^. Although several studies have reported the detailed mechanism of the movement of virions or vRNP through PD^19,23–25^, the active role of MP in this movement remains unclear. Our sequence analysis (Fig. 1A,B) and atomic structure prediction (Fig. 1C) of MP revealed the presence of a classical putative ATPase motif. The crucial Arginine finger II motif is distantly placed in the atomic model from the Walker A, B, sensor, and arginine finger I motifs, which form a cleft or active site for the binding of ATP. Once ATP occupies its position in the active site, the arginine finger comes into the vicinity of the γ-phosphate for hydrolysis. The unique arrangement of ATP-binding and hydrolysis motifs in the primary and tertiary structure of MP (Fig. 1A–C) indicates the structural similarity of the MP with the members of the well-known ASCE P-loop ATPase family^26^. Interestingly, our phylogenetic data further confirm the parallel evolution of the novel ATPase fold of plant viruses along with the well-studied AAA+ and RecA-type ATPase folds (T. Ranjan, Unpublished data). In the future, we plan to replace the critical amino acid residues of these putative ATPase motifs through site directed mutagenesis, which will further dissect the function of these motifs, especially in terms of active genome translocation through PD. Since the ATPase activity is believed to act as driving force for viral genome transportation, the ability of the purified proteins to hydrolyse ATP was analysed (Fig. 2B). Incubation of MP with ATP resulted in the generation of inorganic Pi in a protein concentration-dependent manner, suggesting that MP hydrolysed the β-γ phosphodiester bond, generating ADP and Pi. When DNA/RNA was added to the reaction mixture, it enhanced ATP hydrolysis (Fig. 2B), suggesting the formation of vRNP complexes during the movement of viral genomes across PD.

In the present study, the efficiency of MP siRNA constructs to silence and inhibit PLRV multiplication was assessed. The siRNA constructs were designed against MP of PLRV and agroinfiltrated into infected potato plants. The agroinfiltrated plants did not show PLRV infection. Suppression of viral infection could be attributed to the reduced expression of MP due to its silencing by the siRNA constructs. The present study indicated that the transient expression of MP constructs resulted in specific and efficient inhibition of PLRV. Northern blotting, RT-PCR and ELISA analysis also confirmed that the PLRV MP mRNAs were not detected in the leaves of plants agroinfiltrated with MP siRNA, whereas these mRNAs were detected at high levels in tertiary leaves of control plants (naturally infected) and those agroinfiltrated with the empty vector. These findings further confirm the earlier reports and suggest that MP has a direct role in the active cell-to-cell transportation of the viral genome and virions and is thus involved in systemic translocation^27^.

To the best of our knowledge, this is the first study that presents a novel ATPase fold of MP and demonstrates the suppression of MP expression using siRNA constructs, resulting in the development of resistance against PLRV. The inhibition of the expression of MP by gene silencing is an efficient and promising method to introduce resistance to PLRV^28^. As PLRV causes severe crop yield losses in the potato growing regions worldwide, our findings will be helpful for developing PLRV-resistant potato crops. This approach can also be applied to an extensive range of plant species against various viral diseases. Further, we are developing strategies for the development of PLRV resistant potato varieties using the RNAi approach.

## Supplementary Information

Supplementary Figure S1.

Supplementary Table S1.

Supplementary captions.
