# Supplementary figures and images for: Inhibition of potato leafroll virus multiplication and systemic translocation by siRNA constructs against putative ATPase fold of movement protein
Source: Sci Rep. 2020 Dec 16;10:22016. doi: 10.1038/s41598-020-78791-4 (PMC7744510; doi:10.1038/s41598-020-78791-4)

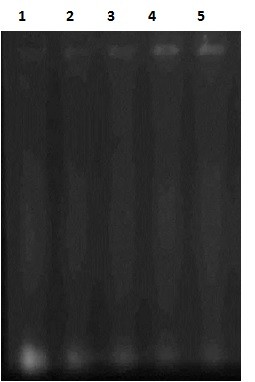

Supplement: Supplementary file 1 — Supplementary Figure S1. [file 41598_2020_78791_MOESM1_ESM.jpg]
