# Supplementary material for: Inhibition of potato leafroll virus multiplication and systemic translocation by siRNA constructs against putative ATPase fold of movement protein
Source: Sci Rep. 2020 Dec 16;10:22016. doi: 10.1038/s41598-020-78791-4 (PMC7744510; doi:10.1038/s41598-020-78791-4)
Supplement: Supplementary file 2 — Supplementary Table S1. [file 41598_2020_78791_MOESM2_ESM.docx]

| **siRNA sequence (5’-3’)** | **Biotin-labeled probe sequence**  **(Complementary siRNA sequence) (5’-3’)** |
| --- | --- |
| UACUCAAGGCCUACCAUGA | UTGUGTTCCGGUTGGTUCT |
| GGCCUACCAUGAGUAUAAG | CCGGUTGGTUCTCUTUTTC |
| AUCGCCGCUCAAGAAGAAC | TUGCGGCGUGTTCTTCTTG |
| AGGAUGGAAUACUCAAGGC | TCCTUCCTTUTGUGTTCCG |
| GGAUGGAAUACUCAAGGCC | CCTUCCTTUTGUGTTCCGG |
| GUAUAAGAUCACAAGCAUC | CUTUTTCTUGTGTTCGTUG |
| UUGUAAACACGAAUGUCUCGC | UUCUTTTGTGCTTUCUGUGCG |
| AAGUAAGAUGCUUGUGAUCUU | TTCUTTCTUCGUUCUCTUGUU |
| UAAUUUGGAACUUGUUGACGU | UTTUUUCCTTGUUCUUCTGCU |

**Inhibition of potato leafroll virus multiplication and systemic translocation by siRNA constructs against putative ATPase fold of movement protein**

^*^Priyanka Kumari^1^, ^*^Jitesh Kumar^1^, Ravi Ranjan Kumar^1^, Mohammad Ansar^2^, Kumari Rajani^3^, Sunil Kumar^4^ and Tushar Ranjan^1#^
