# Supplementary material for: Inhibition of potato leafroll virus multiplication and systemic translocation by siRNA constructs against putative ATPase fold of movement protein
Source: Sci Rep. 2020 Dec 16;10:22016. doi: 10.1038/s41598-020-78791-4 (PMC7744510; doi:10.1038/s41598-020-78791-4)
Supplement: Supplementary file 3 — Supplementary captions. [file 41598_2020_78791_MOESM3_ESM.docx]

**Figure S1:** The DNA binding activity of purified MP. DNA binding activity is increasing with increase in concentration of MP (0.5 – 10 μM). Lane 1: only DNA, lane 2: DNA+MP (0.5 μM), lane 3: DNA+MP (2 μM), lane 4: DNA+MP (4 μM), lane 5: DNA+MP (10 μM).

**Table S1:** Details of biotin-labeled probe sequences used for Northern blotting analysis.
